# Review of Causal Discovery Methods Based on Graphical Models

**DOI:** 10.3389/fgene.2019.00524

**Published:** 2019-06-04

**Authors:** Clark Glymour, Kun Zhang, Peter Spirtes

**Affiliations:** Department of Philosophy, Carnegie Mellon University, Pittsburgh, PA, United States

**Keywords:** directed graphical causal models, causal discovery, conditional independence, statistical independence, structural equation models, non-Gaussian distribution, non-linear models

## Abstract

A fundamental task in various disciplines of science, including biology, is to find underlying causal relations and make use of them. Causal relations can be seen if interventions are properly applied; however, in many cases they are difficult or even impossible to conduct. It is then necessary to discover causal relations by analyzing statistical properties of purely observational data, which is known as causal discovery or causal structure search. This paper aims to give a introduction to and a brief review of the computational methods for causal discovery that were developed in the past three decades, including constraint-based and score-based methods and those based on functional causal models, supplemented by some illustrations and applications.

## 1. Introduction

Almost all of science is about identifying causal relations and the laws or regularities that govern them. Since the seventeenth century beginnings of modern science, there have been two kinds of procedures, and resulting kinds of data, for discovering causes: manipulating and varying features of systems to see what other features do or do not change; and observing the variation of features of systems without manipulation. Both methods shone in the seventeenth century, when they were intertwined then as they are today. Evangelista Torricelli manipulated the angles and shapes of tubes filled with mercury standing in a basin of the stuff, showing the height of the mercury in the tubes did not vary; Pascal had a manometer of Torricelli's design carried up a mountain, the Puy de Dome, to show that the height of the mercury did vary with altitude. Galileo, for whom Torricelli worked, had identified (qualitatively) the orbits of Jovian satellites from observational time series, and similarly characterized sunspots. Kepler, Galileo's northern contemporary, adduced his three laws from planetary observations, and a generation later Newton laid the foundations of modern physics with a gravitational law adduced from solar system observations and a single experiment, on pendulums. Modern molecular biology is an experimental subject, but the foundation of biology, in Darwin's Origin of Species, has only a single experiment, the drifting of seeds.

This paper is about the scientific application of a kind of representation of causal relations, directed graphical causal models (DGCMs), and computerized methods for finding true causal representations of that kind from data, whether observational or experimental or both. We focus on it here because while apparently first proposed in 2000 for studies of gene expression (Murphy and Mian, 1999; Friedman et al., 2000; Spirtes et al., 2000), the models have found wide use in systems biology, especially in omics and in neural connectivity studies, and there has recently been an explosion in the number of algorithms that have been proposed and applied for discovering such representations in biological applications.

A traditional way to discover causal relations is to use interventions or randomized experiments, which is in many cases too expensive, too time-consuming, or even impossible. Therefore, revealing causal information by analyzing purely observational data, known as causal discovery, has drawn much attention (Spirtes et al., 2000). Past decades have seen a series of cross-disciplinary advances in algorithms for identifying causal relations and effect sizes from observational data or mixed experimental and observational data. These developments promise to enable better use of appropriate “big data." They have already been applied in genomics, ecology, epidemiology, space physics, clinical medicine, neuroscience, and many other domains, often with experimental or quasi-experimental validation of their predictions. Causal discovery will be the focus of this review. In traditional causality research, algorithms for identification of causal effects, or inferences about the effects of interventions, when the causal relations are completely or partially known, address a different class of problems; see Pearl (2000) and references therein.

We will start with the so-called constraint-based as well as score-based methods for causal discovery. Since the 1990's, conditional independence relationships in the data have been exploited to recover the underlying causal structure. Typical (conditional independence) constraint-based algorithms include PC and Fast Causal Inference (FCI) (Spirtes et al., 2000). PC assumes that there is no confounder (unobserved direct common cause of two measured variables), and its discovered causal information is asymptotically correct. FCI gives asymptotically correct results even in the presence of confounders. Such approaches are widely applicable because they can handle various types of data distributions and causal relations, given reliable conditional independence testing methods. However, they do not necessarily provide complete causal information because they output (independence) equivalence classes, i.e., a set of causal structures satisfying the same conditional independences. The PC and FCI algorithms produce graphical representations of these equivalence classes. In cases without confounders, there also exist score-based algorithms that aim to find the causal structure by optimizing a properly defined score function. Among them, Greedy Equivalence Search (GES) (Chickering, 2003) is a well-known two-phase procedure that directly searches over the space of equivalence classes.

Recently it has been shown that algorithms based on properly defined Functional Causal Models (FCMs) are able to distinguish between different Directed Acyclic Graphs (DAGs) in the same equivalence class. This benefit is owed to additional assumptions on the data distribution than conditional independence relations. A FCM represents the effect variable *Y* as a function of the direct causes *X* and some noise term *E*, i.e., *Y* = *f*(*X, E*), where *E* is independent of *X*. Thanks to the constrained functional classes, the causal direction between *X* and *Y* is identifiable because the independence condition between the noise and cause holds only for the true causal direction and is violated for the wrong direction. We will review causal discovery methods based on linear non-Gaussian models (Shimizu et al., 2006) or non-linear models (Hoyer et al., 2009; Zhang and Hyvärinen, 2009b), and discuss their applicability.

In practice, for reliable causal discovery one needs to address specific challenges that are often posed in the causal process or the sampling process to generate the observed data. Therefore, we will discuss how to deal with a number of such practical issues, which include causality in time series, measure error, missing data, non-stationarity or heterogeneity of the data, and selection bias. We finally briefly discuss the applications of causal search algorithms as well as some related methods in biology and offer some guidance for their choice and use.

## 2. Directed Graphical Causal Models

A DGCM has the following components: (1) a set of variables, regarded as “random variables," (2) a set of directed edges between pairs of variables, each edge regarded as the hypothesis that the two variables would be associated if all other variables were fixed at some values while the tail variable is exogenously varied, and (3) a joint probability distribution over the possible values of all of the variables. The variables can be time indexed, forming a set of causally related stochastic processes; some of the variables can be unmeasured; the variables can be categorical, ordinal, or continuous; there can be measurement error and selection bias, also graphically represented; and there can be (and are usually assumed to be) omitted sources of variation specific to each variable, often deemed “noise” or “disturbances.” A class of DGCMs, commonly presented as “structural equation models” (SEMs), or functional causal models (FCMs), assumes the value of each variable is a deterministic function of its direct causes in the graph and the unmeasured disturbances. The function linking a variable to its direct causes can be any whatsoever, although linear models are most common. The class of DGCMs includes, but is more general than, regression models, factor models, ARM time series models, latent class models, and others. Requiring neither initial conditions (except in time series) nor boundary conditions, DGCMs contrast with differential and partial differential systems of equations, which can also be representations of a system of causal relations.

Note that not all directed graphical models have causal interpretations–traditional graphical models provide a compact, yet flexible, way to decompose the joint distribution of the data as a product of simpler factors (Koller and Friedman, 2009), and the second component of a DGCM given above is essential for a directed graph to have a causal meaning. It states that two variables with an edge in between are associated if all other variables were fixed at some values while the tail variable is exogenously varied and, hence, indicates that if *X*_*i*_ → *X*_*j*_ in the directed graph, then *X*_*i*_ is a direct cause of *X*_*j*_. In other words, it says that if *X*_*i*_ → *X*_*j*_ in the directed graph, then there exist interventions on *X*_*i*_ that will directly change the distribution (or value) of *X*_*j*_. The causal Bayesian network was defined in a similar way by Pearl (2000, p.23). The pairing of a directed graph and a joint probability distribution on values of its variables is subject to constraints. In the case of a directed graph without cycles (no closed directed paths) the constraint is that a graphical condition–*d-separation*–must imply conditional independence in the probability distribution.

A *path* from a vertex *X*_1_ to a vertex *X*_*n*_ is a sequence of distinct vertices <*X*_1_, …, *X*_*n*_> such that for each pair of vertices *X*_*i*_ and *X*_*i*+1_, there is an edge *X*_*i*_ → *X*_*i*+1_ or *X*_*i*+1_ → *X*_*i*_. A *directed path* from *X*_*i*_ to *X*_*n*_ is a path in which for each pair *X*_*i*_ and *X*_*i*+1_, *X*_*i*_ → *X*_*i*+1_. A variable *X*_*i*_ is a collider on a path *P* iff the path contains *X*_*i*−1_ → *X*_*i*_ ← *X*_*i*+1_ (i.e., *X*_*i*_ is a common effect of its neighbors on the path); otherwise it is a non-collider. For three disjoint sets of variables *X*, *Y*, and *S*, *X* is d-separated from *Y* conditional on *S* iff all paths between any member of *X* and any member of *Y* are blocked by *S*. The path *P* is blocked by *S* if 1) any non-collider on *P* is in *S* *or* if 2) *P* contains a collider which is not in *S* and whose descendants are not in *S*, either.

The graphical property of *d*-separation and its connection with conditional independence has a more intuitive but less practically useful equivalent in the local Markov Condition: every variable, *X*, in a directed acyclic graph, is independent of its non-descendants conditional on its parents (the variables with edges directed into *X*). The Markov condition can be thought of as a generalization of a familiar principle in experimental inference: fixing the values of variables that directly influence some variable of interest, *X*, “screens off” more remote causes that can only influence *X* via the more direct causes. Graphs that have the same *d*-separation properties are usually called “Markov equivalent” and imply the same conditional independence relations; a collection of all directed acyclic graphs that are Markov equivalent is a Markov Equivalence Class (MEC). For linear systems, the graphical property of *d*-separation has been generalized to directed graphs with cycles–closed directed paths (Spirtes, 1995). For a system that has a graph that represents the marginalized graph of a larger system, there is a corresponding relation, *m*-separation (Ayesha et al., 2009). When the Markov condition is assumed to hold for a causal graph and its associated population distribution, it is called the *Causal Markov Assumption*.

It is important to note that d-separation and related properties provide necessary but not sufficient conditions for conditional independence relations in the joint probability distribution over the values of the variables. The probability distribution may have additional conditional independence relations that are not entailed by d-separation applied to a graph. When no such extra conditional independence relations hold the distribution is said to be faithful to the graph (and when assumed to be true of the causal graph and its corresponding population distribution is called the *Causal Faithfulness Assumption*).

The reason for regarding the graphical relations in a DGCM as causal claims, not just a representation of associations or dependence, is that a DGCM entails claims about the results of many hypothetical experiments: if an acyclic DGCM contains a directed edge *X* → *Y*, the experimental claim is that if every other variable represented in the graph is held fixed, *X* and *Y* will covary if *X* is forced to vary, but not if *Y* is forced to vary. These experimental predictions can be computed from the graph and the probability distribution (Spirtes et al., 2001).

## 3. Traditional Constraint-Based and Score-Based Causal Discovery Methods

Roughly speaking, causal search methods are nothing but statistical estimation of parameters describing a graphical causal structure. It is computationally intensive estimation, but statistical estimation of parameters nonetheless, and so understood, something familiar. Most statistical estimators give a number or an interval, the estimated correlation, directly as a function of the data. But other estimators are more laborious. In all but simple models, the estimate of a posterior probability distribution, for example, or the estimate of a cyclic structural equation model usually requires iterative or Monte Carlo procedures, sometimes explicitly described as “searches” (Hoff, 2009).

The parameters to be estimated in the simple case of an acyclic model with non-interactive causes and no unobserved confounders (a confounder is an unobservable direct common cause of two observed variables) are just the entries in an *N* × *N* matrix, where *N* is the number of variables, and an (*i, j*)th entry indicates whether variable *j* is the parent of variable *i*. When “latent” variables are allowed, two possible values for an entry are needed, one indicating a direct connection, the other indicating a confounding by an unobserved common cause. A further value can be added when a direct connection between a pair of variables is unknown rather than known to be absent. The issue is how to estimate any of these parameters.

Statistical estimation has various desiderata. Statistical “consistency," that is, under sampling assumptions, the estimates converge in probability or almost surely to the true value; uniform convergence, in which there are probabilistic bounds on the size of errors at finite sample sizes, etc. Graphical causal model search based on the Faithfulness assumption and which conditional independence relations hold has in general only “pointwise" consistency, which does not provide finite sample error probabilities and does not provide confidence intervals for the estimated structure; although in sequences of models in which the number of variables and sparsity of the graph is controlled as a function of the sample size, there is a uniform consistency result when assumptions stronger than Faithfulness are made (Kalisch and Bühlmann, 2007).

For the most part, there are two classes of search algorithms, and their sub-classes and “nearby methods.” One class of search algorithms tries to efficiently search for a MEC of graphs that most closely entails (under the Causal Markov and Faithfulness Assumptions) the set of conditional independence relations judged to hold in the population. Another class of algorithms estimates the dependencies or conditional independencies of each variable on independent noises, and uses these relations to construct a directed graphical model. We will illustrate how each of these is possible, and mention some variants.

### 3.1. The PC Algorithm

One of the oldest algorithms that is consistent under i.i.d. sampling assuming no latent confounders is the PC algorithm (Spirtes et al., 2001), which provides a search architecture into which can be plugged many statistical procedures for deciding conditional independence. Suppose then we have some such statistical decision procedure, which might be a hypothesis test for conditional independence, or a method based on the difference of fitting scores such as the Bayesian Information Criterion (BIC) between models with and without a particular directed edge.

**Figure 1:**
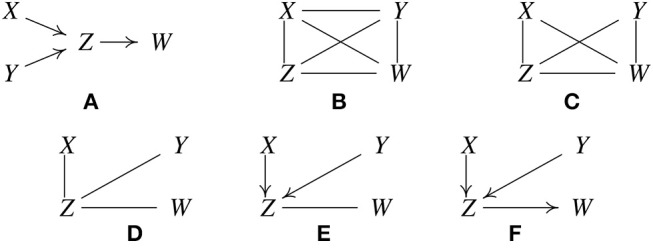
Illustration of how the PC algorithm works. **(A)** Original true causal graph. **(B)** PC starts with a fully-connected undirected graph. **(C)** The *X* − *Y* edge is removed because *X* ⫫ *Y*. **(D)** The *X* − *W* and *Y* − *W* edges are removed because *X* ⫫ *W* |*Z* and *Y* ⫫ *W* |*Z*. **(E)** After finding v-structures. **(F)** After orientation propagation.

Let the true structure be as in Figure 1A. By *d*-separation, this structure implies that *X* is independent of *Y*, written *X* ⫫ *Y*, and that *X* and *Y* are each independent of *W* conditional on *Z*, written {*X, Y*} ⫫ *W*|*Z*. Suppose when called, the statistical decision procedure finds these relations. PC is based on the fact that under the causal Markov condition and the faithfulness assumption, when there is no latent confounder, two variables are directly causally related (with an edge in between) if and only if there does not exist any subset of the remaining variables conditioning on which they are independent (Spirtes et al., 2001). It works like this:

1. Form a complete undirected graph, as in Figure 1B.
2. Eliminate edges between variables that are unconditionally independent; in this case that is the *X* − *Y* edge, giving the graph in Figure 1C.
3. For each pair of variables (*A, B*) having an edge between them, and for each variable *C* with an edge connected to either of them, eliminate the edge between *A* and *B* if *A* ⫫ *B* | *C* as in Figure 1D.
4. For each pair of variables *A, B* having an edge between them, and for each pair of variables {*C, D*} with edges both connected to *A* or both connected to *B*, eliminate the edge between *A* and *B* if *A* ⫫ *B* | {*C, D*}.

Continue checking independencies conditional on subsets of variables of increasing size *n* until there are no more adjacent pairs (*A, B*), such that there is a subset of variables of size *n* such that all of the variables in the subset are adjacent to *A* or all adjacent to *B*. In the considered example, *Z* and *W* are not independent conditional on *X* or on *Y* or on both *X* and *Y*, so there are no further statistical decisions to make. Similarly for *X* and *Z*, and for *Y* and *Z*.

5. For each triple of variables (*A, B, C*) such that *A* and *B* are adjacent, *B* and *C* are adjacent, and *A* and *C* are not adjacent, orient the edges *A* − *B* − *C* as *A* → *B* ← *C*, if *B* was not in the set conditioning on which *A* and *C* became independent and the edge between them was accordingly eliminated. We call such a triple of variables *a v-structure*.

In the example, *Z* was not conditioned on in eliminating the *X*−*Y* edge, so orient *X*−*Z*−*Y* as *X* → *Z* ← *Y*, with the result given in Figure 1E.

6. For each triple of variables such that *A* → *B*−*C*, and *A* and *C* are not adjacent, orient the edge *B*−*C* as *B* → *C*. This is called *orientation propagation*.

In Figure 1F, *Y* → *Z*−*W* is oriented as *Y* → *Z* → *W*. In this example, the true structure is recovered uniquely.

There are several other simple orientation propagation rules that are not illustrated here. The inference steps illustrated are not tuned for the example; they are instances of a general set of rules that hold for any i.i.d. data from a directed acyclic graph. If the conditional independence decisions are correct in the large sample limit, the PC algorithm is guaranteed to converge to the true Markov Equivalence Class in the large sample limit, assuming the Causal Markov and Faithfulness assumptions, i.i.d. samples, and no unmeasured confounders. Note that in some examples, none of orientation rules will apply to a given undirected edge, and that edge will remain undirected in the output. This means that while the two variables are known to be adjacent, it is not known which direction the edge points, or equivalently, there are two different members of the MEC which differ in the direction of that edge. The graphical object with a mixture of directed and undirected edges is called a pattern or CPDAG (Completed Partially Directed Acyclic Graph) that represents a MEC of DAGs. For sparse graphs, the PC algorithm is feasible on at least tens of thousands of variables (in the linear or multinomial case, in which conditional independence test is computationally efficient).

It is worth noting that the output of causal discovery algorithms such as PC is typically different from and much more informative than the so-called “conditional independence graph" (Lauritzen, 1996), in which two variables are not adjacent if and only if they are conditionally independent given all the remaining variables. (The conditional independence graph reduces to the “partial correlation graph" in the special case of jointly Gaussian variables.) In conditional independence graphs, edges are undirected, so they do not have a causal interpretation. Furthermore, the adjacencies may be different from the estimated causal graph; for instance, in the above example, *X* and *Y*, although marginally independent, are not conditionally independent given the rest of the variables, i.e., {*Z, W*}. As a consequence, in the conditional independence graph they will be adjacent, different from the causal graph.

### 3.2. The FCI Algorithm

**Figure 2:**
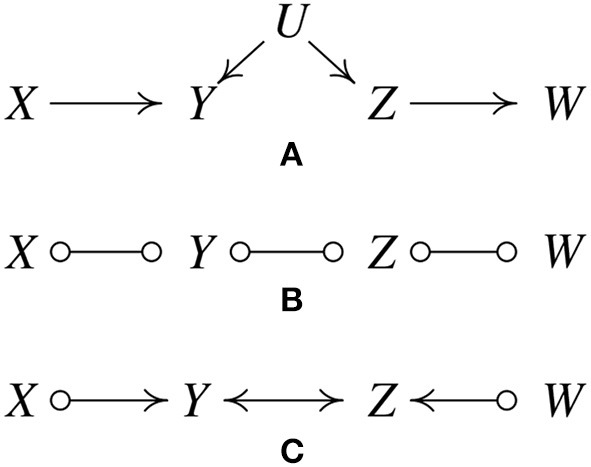
IIllustration of how the FCI algorithm is able to determine the existence of latent confunders. **(A)** Original true causal graph. **(B)** After edges are removed because of conditional independence relations. **(C)** The output of FCI, indicating that there is at least one unmeasured confounder of *Y* and *Z*.

Since its inception, a large number of variations of the PC algorithm have been published and it has been supplemented with a variety of heuristics, or “wrappers.” The most important generalization is the Fast Causal Inference (FCI) Algorithm (Spirtes et al., 2001), which tolerates and sometimes discovers unknown confounding variables. Its results have been shown to be asymptotically correct even in the presence of confounders. Figure 2A, where *U* is an unmeasured variable, illustrates how this is possible without illustrating the full complexity of the FCI algorithm.

As with the first state of the PC procedure, FCI calls statistical independence judgements to prune an undirected graph, yielding Figure 2B.

The “o” mark means it can be an arrow head or an arrow tail. The reason for the “o" marks will become apparent. FCI orients edges by a procedure similar to PC but without assuming that every edge is directed one way or the other. The *X ⧟ Z* edge was eliminated without conditioning on *Y* because *X* and *Z* are unconditionally independent; the *X ⧟ Y ⧟ Z* triple is therefore oriented as a “collider", *X* ⟜ > *Y* < ⊸ *Z*. In the same way, *Y ⧟ Z ⧟ W* is found to be a collider, *Y ⟜* > *Z* < ⊸*W*, yielding Figure 2C.

The bidirected edge between *Y* and *Z* indicates that there is at least one unmeasured confounder of *Y* and *Z*. The remaining “o" symbols at *X* and *W* indicate that the algorithm cannot tell whether the *X, Y* connection is a directed edge from *X* to *Y*, or an unmeasured confounder, or both; the same for the *Z* and *W*. In fact, no algorithm based entirely on conditional independence relations can determine which of these is the case.

In contrast to this example, in which one can determine that there is at least one unmeasured confounder of *Y* and *Z*, there are other situations in which one can exclude the possibility of having confounders. For instance, consider the causal graph in Figure 1A and suppose we have enough data generated by it. Then in the output of FCI, we know that there cannot be any confounder of *Z* and *W*, because otherwise *X* and *W* cannot be independent conditioning on *Z* (*X* and *W* are not *d*-separated by *Z* if *Z* and *W* have a confounder).

As with PC there are variants of FCI, mostly designed to speed up the algorithm at the cost of reduced information (e.g., see the RFCI algorithm Colombo et al., 2012).

### 3.3. The Greedy Equivalence Search Architecture

Instead of beginning with a complete undirected graph, as in PC and FCI, the Greedy Equivalence Search (GES) (Chickering, 2003) starts with an empty graph, and adds currently needed edges, and then eliminates unnecessary edges in a pattern. At each step in the algorithm as decision is made as to whether adding a directed edge to the graph will increase fit measured by some quasi-Bayesian score such as BIC, or even by the *Z* score of a statistical hypothesis test, the edge that most improves fit is added. The resulting model is then mapped to the corresponding Markov equivalence class, and the procedure continued. When the score can no longer be improved, the GES algorithm then asks, edge by edge, which edge removal, if any, will most improve the score, until no further edges can thus be removed. The GES procedure is not so easy to illustrate as is PC, because its trajectory depends on the relative strengths of the associations and conditional associations of the variables. In the large sample limit, however, the two algorithms converge on the same Markov Equivalence Class under assumptions that are nearly the same. On finite samples, the algorithms may give different results, and there is as yet no GES style algorithm for cases with unknown confounders. GFCI (Ogarrio et al., 2016), a combination of GES and FCI, using GES to find a supergraph of the skeleton and FCI to prune the supergraph of the skeleton and find the orientations. GFCI has, however, proved more accurate in many simulations than the original FCI algorithm.

## 4. Non-Gaussian or Non-linear Methods Based on Functional Causal Models

Constraint-based methods for causal discovery involve conditional independence tests, which would be a difficult task if the form of dependence is unknown. It has the advantage that it is generally applicable, but the disadvantages are that faithfulness is a strong assumption and that it may require very large sample sizes to get good conditional independence tests. Furthermore, the solution of this approach to causal discovery is usually non-unique, and in particular, it does not help in determining causal direction in the two-variable case, where no conditional independence relationship is available.

What information can we use to fully determine the causal structure? A fundamental issue is given two variables, how to distinguish cause from effect. To do so, one needs to find a way to capture the asymmetry between them. Intuitively, one may think that the physical process that generates effect from cause is more natural or simple in some way than recovering the cause from effect. How can we represent this generating process, and in which way is the causal process more natural or simple than the backward process?

When talking about the causal relation between two variables, traditionally people were often concerned with the linear-Gaussian case, where the involved variables are Gaussian with a linear causal relation, or the discrete case. It turned out that the former case is one of the atypical situations where the causal asymmetry does not leave a footprint in the observed data or their joint distribution, as explained later in this section.

Recently several causal discovery approaches based on Functional Causal Models (FCMs) have been proposed for causal discovery from continuous variables. A FCM represents the effect *Y* as a function of the direct causes *X* and some unmeasurable factors or noise:

(1) $$\begin{array}{r} Y=f\left(X,\varepsilon ;\theta_{1}\right), \end{array}$$

where ε is the noise term that is assumed to be independent from *X*, the function f∈F explains how *Y* is generated from *X*, F is an appropriately constrained functional class, and **θ**_1_ is the parameter set involved in *f*. We assume that the transformation from (*X*, ε) to (*X, Y*) is invertible, such that *N* can be uniquely recovered from the observed variables *X* and *Y*.

For convenience of presentation, let us assume that both *X* and *Y* are one-dimensional variables. Without precise knowledge on the data-generating process, the FCM should be flexible enough such that it could be adapted to approximate the true data-generating process; more importantly, the causal direction implied by the FCM has to be identifiable in most cases, i.e., the model assumption, especially the independence between the noise and cause, holds for only one direction, such that it implies the causal asymmetry between *X* and *Y*. Under the above conditions, one can then use FCMs to determine the causal direction between two variables, given that they have a direct causal relationship in between and do not have any confounder: for both directions, we fit the FCM, and then test for independence between the estimated noise term and the hypothetical cause, and the direction which gives an independent noise term is considered plausible. It has been shown that without any further assumption on the function *f*, causal direction is not identifiable because for both directions one can find an independent noise term (Hyvärinen and Pajunen, 1999; Zhang et al., 2015).

Several forms of the FCM have been shown to be able to produce unique causal directions, and have received practical applications. In the linear, non-Gaussian, and acyclic model (LiNGAM) (Shimizu et al., 2006), *f* is linear, and at most one of the noise term ε and cause *X* is Gaussian. In the post-nonlinear (PNL) causal model (Zhang and Chan, 2006; Zhang and Hyvärinen, 2009b), the effect *Y* is further generated by a post-nonlinear transformation on the non-linear effect of the cause *X* plus noise term ε:

(2) $$\begin{array}{r} Y=f_{2}\left(f_{1}\left(X\right)+\varepsilon \right), \end{array}$$

where both *f*_1_ and *f*_2_ are non-linear functions and *f*_2_ is assumed to be invertible.^1^ The post-nonlinear transformation *f*_2_ represents sensor or measurement distortion, which is frequently encountered in practice. In particular, the PNL causal model has a very general form (LiNGAM is clearly a special case), but it has been shown to be identifiable in the generic case [except five specific situations given in Zhang and Hyvärinen (2009b)]. Another special case of the PNL causal model, the non-linear additive noise model (Hoyer et al., 2009; Zhang and Hyvärinen, 2009a) assumes that *f* is non-linear with additive noise ε, i.e., that *f*_2_ in Equation 2 is the identity mapping. Below we will discuss the identifiability of causal direction according to various FCMs, how to distinguish cause from effect with the FCM, and the applicability of causal discovery methods based on those FCMs.

It is worth noting that in the discrete case, if one knows precisely what FCM class generated the effect from cause, which, for instance, may be the noisy AND or noisy XOR gate, then under mild conditions the causal direction can be easily seen from the data distribution. However, generally speaking, if the precise functional class of the causal process is unknown, in the discrete case it is difficult to recover the causal direction from observed data, especially when the cardinality of the variables is small. As an illustration, let us consider the situation where the causal process first generates continuous data and discretizes such data to produce the observed discrete ones. It is then not surprising that certain properties of the causal process are lost due to discretization, making causal discovery more difficult. In this paper we mainly focus on the continuous case. Readers who are interested in causal discovery from discrete variables or mixed discrete and continuous variables may refer to Peters et al. (2010); Cai et al. (2018); Huang et al. (2018).

### 4.1. Method Based on the Linear, Non-gaussian Model

**Figure 3:**
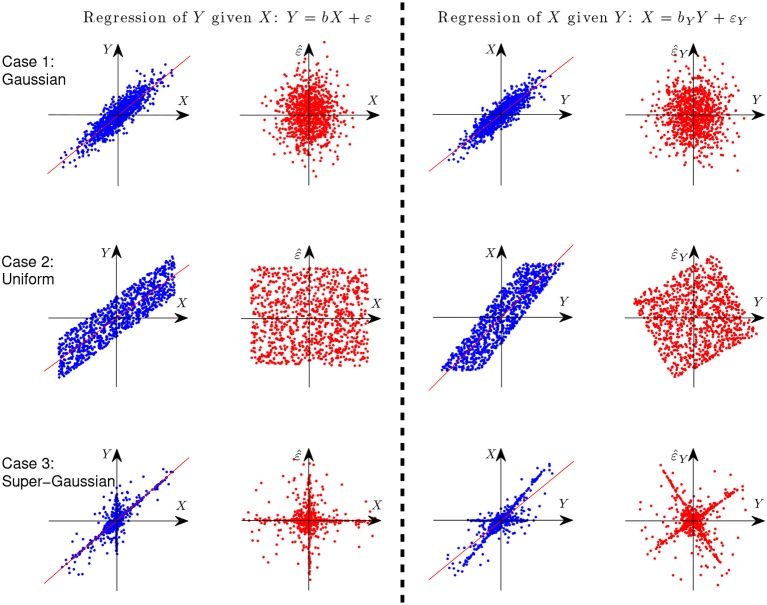
Illustration of causal asymmetry between two variables with linear relations. The causal relation is *X* → *Y*. From top to bottom: *X* and *E* both follow the Gaussian distribution (case 1), uniform distribution (case 2), and Laplace distribution (case 3). The two columns on the left show the scatter plot of *X* and *Y* and that of *X* and the regression residual for regressing *Y* on *X*, and the two columns on the right correspond to regressing *X* on *Y*.

The linear causal model in the two-variable case can be written as:

(3) $$\begin{array}{r} Y=bX+\varepsilon , \end{array}$$

where ε ⫫ *X*. Let us first give an illustration with simple examples why it is possible to identify the causal direction between two variables in the linear case. Assume *Y* is generated from *X* in a linear form, i.e., *Y* = *X*+ε, where ε ⫫ *X*. Figure 3 gives the scatterplot of 1,000 data points of the two variables *X* and *Y* (columns 1 and 3) and that of the predictor and regression residual for two different regression tasks (columns 2 and 4). The three rows correspond to different settings: *X* and *E* are both Gaussian (case 1), uniformly distributed (case 2), and distributed according to some super-Gaussian distribution (case 3). In the latter two settings, *X* and *E* are non-Gaussian, and one can see clearly that for regression of *X* given *Y* (the anti-causal or backward direction), the regression residual is not independent from the predictor anymore, although they are uncorrelated by construction of regression. In other words, in those two situations the regression residual is independent from the predictor only for the correct causal direction, giving rise to the causal asymmetry between *X* and *Y*.

Rigorously speaking, if at most one of *X* and ε is Gaussian, the causal direction is identifiable, due to the independent component analysis (ICA) theory (Hyvärinen et al., 2001), or more fundamentally, due to the Darmois-Skitovich theorem (Kagan et al., 1973). This is known as the LiNGAM (Shimizu et al., 2006).

LiNGAM can be estimated from observational data in a computationally relatively efficient way. Suppose we aim to estimate the causal model underlying the observable random vector $\text{X}={\left(X_{1},\ldots ,X_{n}\right)}^{⊺}$. (Note that here we abuse notation slightly by using **X** as a vector of random variables and *X*_*i*_ as a random variable, while *X* denoted a random variable above.) In matrix form we can represent such causal relations with a matrix **B**, i.e., **X** = **BX**+**E**, where **B** can be permuted to a strictly lower-triangular matrix and **E** is the vector of independent error terms. This can be rewritten as:

(4) $$\begin{array}{r} \text{E}=\left(\text{I}-\text{B}\right)\text{X}, \end{array}$$

where **I** denotes the identity matrix. The approach of ICA-LiNGAM (Shimizu et al., 2006) estimate the matrix **B** in two steps. It first applies ICA (Hyvärinen et al., 2001) on the data:

(5) $$\begin{array}{r} \text{Z}=\text{W}\text{X}, \end{array}$$

such that **Z** has independent components. Second, an estimate of **B** can be found by permuting and resealing the matrix **W**, as implied by the correspondence between (Equations 4, 5).

As the number of variables, *n*, increases, the estimated linear transformation **W** may more likely converge to local optima and involve more and more random errors, causing estimation errors in the causal model. Bear in mind that the causal matrix we aim to estimate, **B**, is very sparse because it can be permuted to a strictly lower-triangular matrix. Hence, to improve the estimation efficiency, one may enforce the sparsity constraint on the entries of **W**, as achieved by ICA with sparse connections (Zhang et al., 2009) or the Two-Step method (Sanchez-Romero et al., 2019). Another way to reduce the estimation error is to find the causal ordering by recursively performing regression and independence test between the predictor and residual, as done by DirectLiNGAM (Shimizu et al., 2011).

It is worth mentioning that in the linear case, it is possible to further estimate the effect of the underlying confounders in the system, if there are any, by exploiting overcomplete ICA (which allows more independent sources than observed variables) (Hoyer et al., 2008). Furthermore, when the underlying causal model has cycles or feedbacks, which violates the acyclicity assumption, one may still be able to reveal the causal knowledge under certain assumptions (Lacerda et al., 2008; Sanchez-Romero et al., 2019).

Finally, one may then challenge the non-Gaussianity assumption in the LiNGAM model as well as its extensions. Here we argue that in the linear case, non-Gaussian distributions are ubiquitous. Cramér's decomposition theorem states that if the sum of two independent real-valued random variables is Gaussian, then both of the summand variables much be Gaussian as well; see (Cramér, 1970, page 53). By induction, this means that if the sum of any finite independent real-valued variables is Gaussian, then all summands must be Gaussian. In other words, a Gaussian distribution can never be exactly produced by linear composition of variables any of which is non-Gaussian. This nicely complements the central limit theorem: (under proper conditions) the sum of independent variable get closer to Gaussian, but it cannot be exactly Gaussian, except all summand variables are Gaussian. This linear closure property of the Gaussian distribution implies the rareness of the Gaussian distribution and ubiquitousness of non-Gaussian distributions, if we believe the relations between variables are linear. However, the closer it gets to Gaussian, the harder it is to distinguish the direction. Hence, the practical question is, are the errors typically non-Gaussian enough to distinguish causal directions in the linear case?

### 4.2. Non-linear Methods

In practice non-linear transformation is often involved in the data generating process, and should be taken into account in the functional class. As a direct extension of LiNGAM, the non-linear additive noise model represents the effect as a non-linear function of the cause plus independent error (Hoyer et al., 2009):

(6) $$\begin{array}{r} Y=f_{AN}\left(X\right)+\varepsilon . \end{array}$$

The above model, as well as LiNGAM, enforces rather strong constraints on the causal process. If the assumed FCM is too restrictive to be able to approximate the true data generating process, the causal discovery results may be misleading. Therefore, if the specific knowledge about the data generating mechanism is not available, to make it useful in practice, the assumed causal model should be general enough, such that it can reveal the data generating processes approximately.

The PNL causal model takes into account the non-linear influence from the cause, the noise effect, and the possible sensor or measurement distortion in the observed variables (Zhang and Chan, 2006; Zhang and Hyvärinen, 2009b). See (2) for its form. It has the most general form among all well-defined FCMs according to which the causal direction is identifiable in the general case. (The model used in Mooij et al. (2010) does not impose structural constraints but assumes a certain type of smoothness; however, it does not lead to theoretical identifiability results.) Clearly it contains the linear model and non-linear additive noise model as special cases. The multiplicative noise model, *Y* = *X*·ε, where all involved variables are positive, is another special case, since it can be written as *Y* = exp(log*X*+logε), where logε is considered as a new noise term, *f*_1_(*X*) = log(*X*), and *f*_2_(·) = exp(·).

The identifiability of the causal direction is a crucial issue in FCM-based causal discovery. Since LiNGAM and the non-linear additive noise model are special cases of the PNL causal model, the identifiability conditions of the causal direction for the PNL causal model also entail those for the former two FCMs. Such identifiability conditions for the PNL causal model was established by a proof by contradiction (Zhang and Hyvärinen, 2009b). It assumes the causal model holds in both directions *X* → *Y* and *Y* → *X*, and show that this implies very strong conditions on the distributions and functions involved in the model. Under certain conditions [e.g., *p*(ε) is positive on (−∞, +∞)], there are only all five cases in which the causal direction is *not* identifiable according to the PNL causal model (Zhang and Hyvärinen, 2009b). The first one is the linear-Gaussian case, in which the causal direction is well-known to be non-identifiable. Suppose the data were generated according to the PNL causal model in settings other than those specific conditions; then in principle, the backward direction does not follow the model, and the causal direction can be determined.

Generally speaking, causal discovery based on non-linear FCMs are not computationally as efficient as in the linear case. Non-linear causal models have been used for distinguishing cause from effect given two variables which are believed to be directly causally related (Hoyer et al., 2009; Zhang and Hyvärinen, 2009b; Peters et al., 2017): they are fitted to data in both directions, and the direction in which the estimated noise is independent from the hypothetical cause (or equivalently, the direction with a higher likelihood) is regarded as causal direction. They can be easily combined with conditional independence-based methods (Zhang and Hyvärinen, 2009b): conditional independence-based methods estimate the MEC from observational data with non-linear or non-parametric methods for conditional independence tests (e.g., the kernel-based method Zhang et al., 2011a), and then non-linear models are applied to further orient the undirected edges in the MEC.

## 5. Several Biological Examples

**Figure 4:**
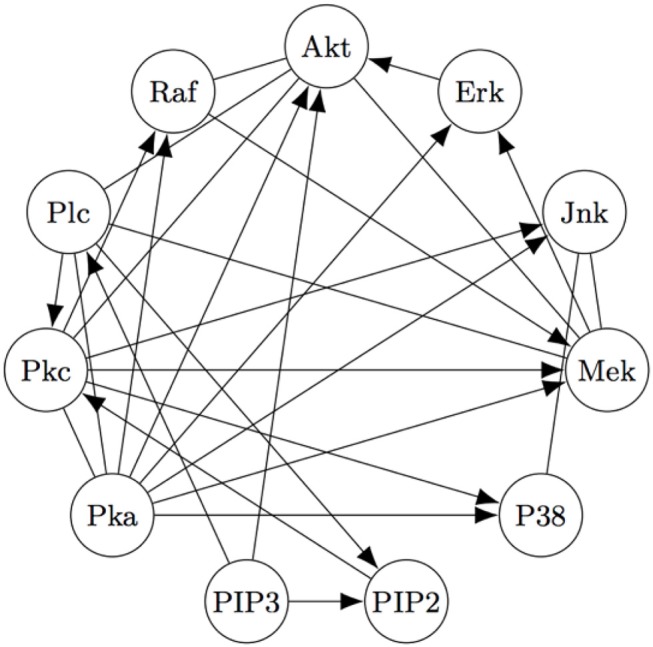
The “extended expert" model for Sachs's data set). See Sachs et al. (2005) or Ramsey and Bryan (2018b) for the significance of the variables.

The Two-Step algorithm and the FASK algorithm (Sanchez-Romero et al., 2019) are two examples of procedures that use adjacency searches to provide an initial undirected directed graph which the algorithms then prune, refine, or extend. Non-Gaussian features of the signal are then used (in different ways) to direct edges, allowing cyclic graphs. Two-Step, but not FASK, also allows for unmeasured confounding. FASK has been applied (Ramsey and Bryan, 2018b) to a famous data set (Sachs's data set Sachs et al., 2005) recording various cellular protein concentrations under a variety of exogenous chemical inputs. Sach's gives an expert model, and in supplementary data gives additional connections for which the experimental literature is not entirely consistent. The data has been reanalyzed several times by various methods, generally not recovering Sach's model or the “expert” model alone or with supplementary edges. Allowing these supplementary edges as undirected edges, the “extended expert" model Ramsey and Andrews use is given in Figure 4.

**Figure 5:**
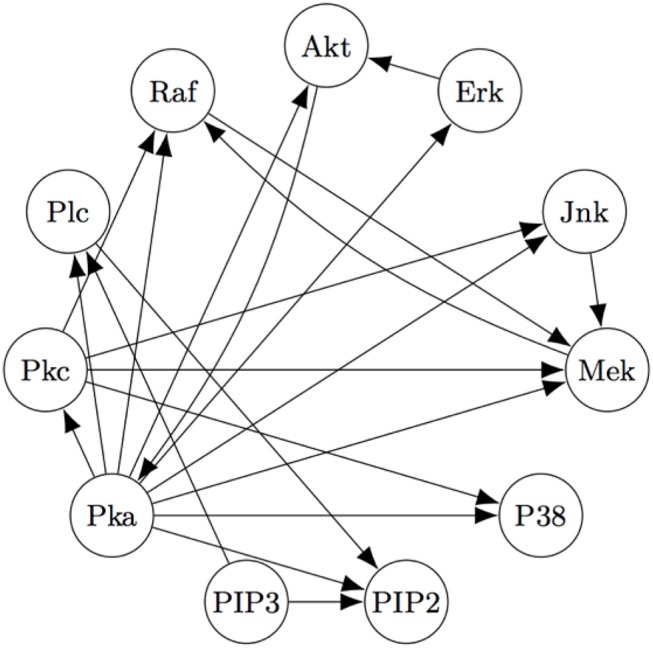
The Model for the Sach's Data estimated by the FASK algorithm.

Using FASK, and using the knowledge that the experimental treatments are exogenous, they recover the model, given in Figure 5, automatically with default values for the search algorithm.

Figure 5 is very close to Figure 4, replacing undirected edges with directed edges or 2-cycles (cycles between only two variables) and supplementing some directed edges with a feedback reciprocity. The most noticeable fault in the FASK model is the absence of the *Mek* → *ERK* edge, which is well-established.

The Causal Stability algorithm is another example of an automated procedure applied to biological data (Stekhoven et al., 2012). The question under consideration was which genes influence the time to flowering of the Arabadopsis thaliana flower. The available data had measurements of expression levels of 21,326 genes for 47 samples from diverse geographic origins. The Causal Stability Ranking algorithm that was used had the following steps. After the data was preprocessed, subsample of size *n*/2 were drawn 100 times. On each subsample, the PC algorithm was run, and then for each gene a lower bound on the absolute value of the total causal effect on time to flowering was estimated by the IDA algorithm, using the output pattern of the PC algorithm and the data as input. (Conceptually, the IDA algorithm estimates the total effect of a gene on time to flowering by estimating the total effect for each DAG represented by the output pattern of PC.) The minimum absolute values of the estimated total effect of each gene on time to flowering were then used to rank the genes. Finally, for a range of different *q* values ({100, 150, 200, …, 2, 000}) the frequency with which each gene appeared in the top *q* genes was calculated. The median rank over the different values of *q* was then used to generate a final ranking of the genes.

When ordered in this way, the top 25 genes contained 5 genes that were known to cause time to flowering. Of the remaining 20 genes that were not known to cause time to flowering, 13 genes had readily available mutants that could be easily tested experimentally to see if the mutant plants differed significantly from the wild type with respect to time to flowering. Of the 13 mutants, 4 had viability issues. Of the 9 remaining genes without viability issues, 4 were novel genes found to cause time to flowering.

## 6. Practical Issues in Causal Discovery

Causal discovery aims to find causal relations by analyzing observational data. The data are produced by not only the underlying causal process, but also the sampling process. In practice, to achieve reliable causal discovery, one needs to address specific challenges posed in the causal process or the sampling process. Below we report some particular issues that have recently been considered; for many of them, better approaches are still needed to improve the reliability and computational efficiency of causal discovery.

### 6.1. Causality in Time Series

Multivariate time series provide the data for many biological and other scientific inquiries, for example mRNA expression series in genomics, and Blood Oxygenation Level Dependent (BOLD) time series in neuropsychology. Finding the causal dynamics generating such data is challenging for many reasons, including that the generating process may be non-linear, the data acquisition rate may be much slower than the underlying rate of changes, there may be measurement error, the system may be non-stationary (i.e., the probability distributions of variables conditional on their causes may change, and even the causal relations may change) and there may be unmeasured confounding causes. The general problem of estimating the causal generating processes for time series is not close to solved, but there is progress in understanding how to deal with these problems in various classes of cases, and increased understanding of why popular methods do not work. In principle, any of the methods described previously, as well as others, can be used on time series. But their accuracies are sensitive to all of the factors just mentioned.

There are several strategies for treating time series data. One is to partition the data into disjoint “windows" and take the measurements in each unit as a data analytic unit. Another is to assume or estimate a number of lagged effects and treat all measurements separated by no more than that number of lags as a data analysis unit. That is a standard procedure in vector autoregression, or what is often called “Granger Causality." A further alternative is to treat the measurements at any time as independent of the measurements at any other time. Each has its disadvantages. The window method necessarily omits relations across windows and results may vary with the choice of window size. In the other two procedures, the units are not all independent, but most units are.

It is well-established that the most common procedure, Granger causality, is very sensitive to temporal aggregation or subsampling [for the effect of aggregation and subsampling and some possible ways to deal with them, see (Danks and Plis, 2013; Gong* et al., 2015; Gong et al., 2017)]. If the sampling rate is equal to the actual time interval required for a signal to propagate (and there is no confounder), Granger's method is very accurate. However, in many times series, data are subsampled or temporally aggregated due to the measuring device or sampling procedure, or for the purpose of efficient collection and storage. It has been shown that under suitable assumptions, the true causal relations are still identifiable from both subsampled and temporally aggregated data; interested readers may refer to Gong* et al. (2015); Gong et al. (2017) and references therein. In particular, It was shown that due to highly temporally aggregated data, time-delayed causal influences in the original causal process appear to be instantaneous in the aggregated time series, which implies that the estimated instantaneous causal relations from low-resolution aggregated data are consistent with the underlying causal influences (Gong et al., 2017).

On the application side, a good deal of research has been done on functional Magnetic Resonance Imaging (fMRI) time series, including a recent comparison of multiple methods. Roughly speaking, one can view fMRI data as some kind of highly temporally aggregated version of the underlying neural activities. The Two-Step and FASK procedures, described in section 5, prove to have the best precision (percentage of edges found that are correct) and recall (percentage of true edges that are found) (Sanchez-Romero et al., 2019). Remarkably, they are robust to (simulated) errors in variables whose variance is no larger than the measurement-free variance of the variables. Two-step retains high precision but has large recall losses; FASK retains both good precision and recall.

### 6.2. Other Issues

Below are some other issues that arise in many causal discovery tasks.

- **Deterministic case**. In a particular deterministic case where *Y* = *f*(*X*) without noise, it is impossible to make use of the independence between noise and the cause to find the causal direction. However, one may exploit a certain type of independence between the transformation *f* and the distribution of the cause *X* to characterize the causal asymmetry and determine the causal direction (Janzing et al., 2012).
- **Nonstationary/heterogenous data**. It is commonplace to encounter nonstationary or heterogeneous data, in which the underlying generating process changes over time or across data sets. Interestingly, if the qualitative causal structure is fixed and the mechanisms or parameters associated with the causal structure may change across data sets or over time (the mechanisms may change such that some causal links in the structure vanish over some time periods or domains), causal discovery may benefit from distribution shift because causal modeling and distribution shift are heavily coupled. This, in particular, inspires a framework for causal mechanism change detection, causal skeleton estimation, causal direction identification, and nonstationary driving force estimation (Huang et al., 2017;Zhang et al., 2017b).
- **Measurement error**. Measurement error in the observed values of the variables can greatly change the output of various causal discovery methods. Given the ubiquity of measurement error caused by instruments or proxies used in the measuring process, this problem has received much attention, and sufficient conditions under which the causal model for the underlying measurement-error-free variables can be partially or completely identified in the presence of measurement error with unknown variance have been established (Zhang et al., 2017a). This will hopefully inspire a set of causal discovery methods dealing with measurement error.
- **Selection Bias**. Selection bias is an important issue in statistical inference, which arises when the probability of including a data point into the sample depends on some attributes of the point. Selection bias, if not corrected, often distorts the results of statistical analysis and causal discovery and inference. In the presence of outcome-dependent selection bias, with FCM-based causal discovery it is possible to identify the correct causal direction and estimate properties of the causal mechanism (Zhang et al., 2016). More general situations with selection bias remain to be studied.
- **Missing data**. Missing data are ubiquitous in many domains such as healthcare. When these data entries are not missing completely at random, the (conditional) independence relations in the observed data may be different from those in the complete data generated by the underlying causal process. Consequently, simply applying existing causal discovery methods to the observed data may lead to the wrong conclusions. A modified PC algorithm was proposed for causal discovery in the presence of missing data (Tu et al., 2019), whose output is asymptotically correct under certain assumptions.

## 7. Application of Causal Discovery in Biology and Some Guidelines to Practice

A great deal of research in biology applies traditional machine learning techniques to various data sets, e.g., genome sequencing data sets (Libbrecht and William, 2015), without trying to find causal relations. In contrast, within the past two decades there has been an increasing number of publications on reconstructing gene regulatory networks or other types of networks in biology; for reviews of this line of work, see e.g., (Narendra et al., 2011; Frolova, 2012; Marbach et al., 2012; Villaverde et al., 2013; Djordjevic et al., 2014; Sinoquet, 2014; Li et al., 2015; Liu, 2015; Hill et al., 2016; Banf and Rhee, 2017). Not all of them aimed to find causal information represented by directed graphs. Many studies tried to derive weaker notions of causal representations, by making use of pairwise dependence between variables (Butte and Kohane, 2001; Margolin et al., 2006), or estimating a partial correlation graph (de la Fuente et al., 2004), or finding a partial correlation graph and further combining it with heuristic methods to infer partial ordering (Opgen-Rhein and Strimmer, 2007), or learning particular types of undirected graph structure (e.g., a tree structure) from data (Huynh-Thu and Sanguinetti, 2015; Gitter et al., 2016), or discovering Markov Blankets of the variables (Ram and Chetty, 2011). Some work infers causal associations between gene expression and disease (Schadt et al., 2005)–luckily, the causal direction between gene expression and disease is known. A number of studies rely on Bayesian network learning to infer certain information of the network; see, e.g., (Pe'er et al., 2001; Auliac et al., 2008;Adabor et al., 2015).

Causal discovery methods or their underlying ideas already received some applications in genetics. For instance, the idea of the PC algorithm was adopted to infer causal relationships among phenotypes (Neto et al., 2010), to estimate gene regulatory networks (Zhang et al., 2011b), and to model the isoprenoid gene network in Arabidopsis thaliana (Wille et al., 2004). Granger causal analysis received a number of applications in estimation of gene regulatory networks; see, e.g., (Michailidis and d'Alché Buc, 2013; Emad and Milenkovic, 2014; Carlin et al., 2017; Yang et al., 2017; Finkle et al., 2018), and similarly, some findings were based on dynamic Bayesian network learning from observational biological data (Yu et al., 2004; Wu and Liu, 2008; Vasimuddin and Srinivas, 2017). There are also applications of network inference methods to leverage multiple data sets (Reiss et al., 2006; Joshi et al., 2015; Zitnik and Zupan, 2015; Omranian et al., 2016). Extended approaches to specific types of network estimation problems also exist, including network deconvolution (Feizi et al., 2013) and network inference by ANOVA (Küffner et al., 2012).

Overall, although the past 30 years witnessed remarkable progress in development of theories and practical methods for automated causal search, they received rather limited applications in biology–in fact, practical causal analysis is not a matter of pressing a few buttons. There are multiple algorithms available, many of them are poorly tested, some of them are poor implementations of good algorithms, some of them are just plain poor algorithms, all of them have choices of parameters, and all of them have conditions on the data distributions and other assumptions under which they will be informative rather than misleading. We offer some general guidelines to practice (see also Malinsky and Danks, 2018).

1. Look at the distributions of the variables. This can be done by visualization or by performing a statistical test. For continuous variables a critical question is whether the distribution is Gaussian or non-Gaussian. This can be checked by an Anderson-Darling Test or eyeballed via a Q-Q plot. If the variables are non-Gaussian, the scatterplots of paired variables can give an indication of whether their relations are linear, polynomial of some obvious kind, periodic, or a mixture of distributions.
2. Check that preprocessing software has not distorted the distributions. For example, standard “high pass" filtering in fMRI software, eliminates some or all of the non-Gaussianity in variables and, as a consequence, the best available algorithms for fMRI time series become uninformative.
3. For continuous variables, check to see if the data are actually mixtures of different causal processes. If the individual causal processes follow linear-Gaussian models, there is a tool–Unmixer–in the TETRAD suite of causal search tools (http://www.ccd.pitt.edu) , and a tool for non-Gaussian variables is in development. If there are a small number of multiple components, the Unmixer algorithm will sort cases; a distinct label as an exogenous variable can be attached to each case, identifying the component to which it belongs, and searches can be run with that additional information. Alternatively, an algorithm such as IMaGES, designed for differing distributions with different linear coefficients, can be run.
4. If the data contain both categorical and continuous variables and there can possibly be categorical variables that are effects and causes, the Conditional Gaussian search in TETRAD can address the problem. That algorithm has limitations suggested by its name: it assumes conditional on values on categorical parents that a continuous variable is Gaussian, and it assumes there are no latent confounders. Work is in progress on generalizations. Alternatively, the continuous variables can be discretized, which sometimes works best when the continuous variables have very non-linear relations. Discretization has a terrible cost in effective sample size, and is only advisable when the number of samples is much larger compared to the number of variables.
5. There is no consensus about what to do about missing values. There is R software for imputing missing values, and commonly used simple strategies such as imputing a mode or median value of a variable, or even deleting an entire case if it has one or more missing values (not recommended). In search algorithms that proceed by evaluating conditional independence on specially chosen subsets of variables (such as the PC algorithm), evaluations can be done simply by ignoring missing values for the relevant variables. It has been shown that this scheme may produce spurious edges (Tu et al., 2019), and an extension of the PC algorithm was also proposed there, whose results are asymptotically correct under certain assumptions on the missingness mechanism.
6. Decide whether the data may suffer from sample selection bias, or measurement error, or unmeasured common causes. If there is sample selection bias and/or unmeasured common causes, there are algorithms, GFCI, FCI, RFCI in the TETRAD suite, and Matlab procedures, Two-Step, that tolerate unknown latent common causes.
7. Specify known influences between measured variables, or known absences of influences. In experimental data, treatment should not be caused by putative effects of treatment. In fMRI studies, for example, stimuli can be convolved with a hemodynamic response function; in biological experiments, the application of a chemical dosage is an exogenous variable. Known exogenous relations can be used to test a search algorithm: they provide a “gold" standard. Further, known causal relations actively guide some search algorithms and result in improved recall and precision.
8. If something about the system is known, test search algorithms and parameter choices on simulated systems that, so far as possible, mimic the observed distributions.
9. There is no consensus about choosing parameter values for search. For undirected graphs estimated by LASSO, there is a cross-validation procedure or BIC for parameter setting. For causal searches using a BIC score there is an adjustable penalty that forces sparsity on the output (in finite samples, of course). Work is in progress on how best to adjust search parameter values (e.g., the significance levels in hypothesis tests) as a function of sample size, number of variables, and sparsity. Search procedures generally do not have confidence intervals for their results, and a “test" of the whole of a high dimensional model seldom makes any sense: many weak dependencies will not be found, but cumulatively they contribute to the real distribution, and so failure of complete recall will result in rejection of a model. Further, in complex models something is wrong somewhere almost always (precision is not perfect), and the model as a whole will typically be rejected by a test. Comparison tests are of course possible–e.g., against a completely disconnected graphical model–but they are not informative about the truth of the selected model.
10. There are very few publicly available competent tests of model search methods. New methods are proposed almost monthly, and published packages vary in quality of implementation. Accuracy recommendations based on public contests are limited to whatever algorithms were submitted to the contests and the particular properties of the data. For DAG searches for linear models (Ramsey and Bryan, 2018a) provides a careful assessment of the most prominent public methods. Bootstrapping an algorithm repeatedly on the data can be informative about how much to trust the output, and can give an estimate of the probabilities of edges . If the results vary widely over the different bootstrap samples, the output should not be trusted. Unfortunately, the converse is not true - stable output is not necessarily correct causal output.

## 8. Conclusion and Discussions

Understanding causal relations is helpful for constructing interventions to achieve certain objectives and also enables making predictions under interventions. It is an important issue in most disciplines of science, especially in biology and neuroscience. A traditional way to discover causal relations is to use interventions or randomized experiments, which is, however, in many cases of interest too expensive, too time-consuming, unethical, or even impossible. Therefore, inferring the underlying causal structure from purely observational data, or from combinations of observational and experimental data, has drawn much attention in various disciplines. With the rapid accumulation of huge volumes of data, it is necessary to develop automatic causal search algorithms that scale well.

We have reviewed two types of causal search algorithms. One makes use of conditional independence relations in the data to find a Markov equivalence class of directed causal structures. Typical algorithms include the PC algorithm, FCI, and the GES algorithm. The other makes use of suitable classes of structural equation models and is able to find a unique causal structure under certain assumptions, for which the condition that noise is independent from causes plays an important role. We have reviewed model classes including LiNGAM, the non-linear additive noise (ANM) model, and the post-nonlinear (PNL) causal model.

Each of the useful methods has its own pros and cons. The PC algorithm and FCI, as typical methods relying on conditional independence relations, require decisions on conditional independence as input, which is straightforward in linear cases (for instance, by Fisher *Z* tests or differences in BIC scores) but rather difficult in general non-linear situations. For linear causal relations, the search procedures can scale very well (e.g., PC and GES can easily deal with tens of thousands of variables for sparse graphs). But on the other hand, their output is a Markov equivalence class, which contains all directed graphs sharing the same conditional independence relations–in this case, the output may not be informative enough in certain circumstances. Methods based on structure equations models have to resort to the functional form of the causal influence, and generally speaking, they cannot handle latent confounders in a straightforward way. The non-Gaussian or non-linear functional causal models help identify more detailed information of the causal process; however, causal search methods based on them usually do not scale as well as those conditional-independence-based methods. To estimate LiNGAM, the estimation methods Two-Step and FASK are feasible on thousands of variables generated by a sparse graph. Current methods for estimating non-linear causal models are feasible on only dozens of variables. Table 1 summarizes the assumptions and properties of the fundamental causal discovery methods reviewed in the paper, as well as a summary of the contributions to address some of the practical issues that often arise in causal discovery in biology, especially in genetics.

**Table 1:** Comparison of the fundamental causal discovery methods reviewed in this paper.

	**PC**	**FCI**	**GES**	**LiNGAM/PNL/ANM**
Faithfulness assumption required?	Yes	Yes	Some weaker condition required (not totally clear yet)	No
Specific assumptions on data distributions required?	No	No	Yes (usually assumes linear-Gaussian models or multinomial distributions)	Yes
Properly handle confounders?	No	Yes	No	No
Output	Markov equivalence class	Partial ancestral graph	Markov equivalence class	DAG as well as causal model (under the respective identifiability conditions)
Remark on practical issues	Confounder in the linear, non-Gaussian case Hoyer et al. (2008); feedback in linear cases Lacerda et al. (2008); Sanchez-Romero et al. (2019); measurement error Zhang et al. (2017a); non-stationary times series or heterogeneous multiple data sets Huang et al. (2017); Zhang et al. (2017b); missing data Tu et al. (2019); subsampled or aggregated time series Danks and Plis (2013); Gong* et al. (2015); Gong et al. (2017), etc.			

Finally, we note that for reliable causal discovery, one often needs to address particular challenges that may be posed in the causal process or in the sampling process to generate the observed data. Typical challenges include sampling bias in the data, various types of non-linear effects, existence of measurement error, confounding effects, and heterogeneity of the data. Better methods to deal with those issues will clearly improve the quality of causal structure search, especially in genetics.

### Some Software Packages and Source Code

The following software packages that are relevant to causal discovery, among others, are available online.

- The Tetrad project webpage (Tetrad implements a large number of causal discovery methods, including PC and its variants, FCI, and LiNGAM):
  http://www.phil.cmu.edu/tetrad/
- Kernel-based conditional independence test (Zhang et al., 2011a):
  http://people.tuebingen.mpg.de/kzhang/KCI-test.zip
- LiNGAM and its extensions (Shimizu et al., 2006, 2011):
  https://sites.google.com/site/sshimizu06/lingam
- Fitting the nonlinear additive noise model (Hoyer et al., 2009):
  http://webdav.tuebingen.mpg.de/causality/additive-noisetar.gz
- Distinguishing cause from effect based on the PNL causal model (Zhang and Hyvärinen, 2009b):
  http://webdav.tuebingen.mpg.de/causality/CauseOrEffect_NICA.rar
- Probabilistic latent variable models for distinguishing between cause and effect (Mooij et al., 2010):
  http://webdav.tuebingen.mpg.de/causality/nips2010-gpi-code.tar.gz
- Information-geometric causal inference (Janzing et al., 2012):
  http://webdav.tuebingen.mpg.de/causality/igci.tar.gz

## Author Contributions

All authors listed have made a substantial, direct and intellectual contribution to the work, and approved it for publication.

### Conflict of Interest Statement

The authors declare that the research was conducted in the absence of any commercial or financial relationships that could be construed as a potential conflict of interest.

## Footnotes

^1^In Zhang and Chan (2006) both functions *f*_1_ and *f*_2_ are assumed to invertible; this causal model, as a consequence, can be estimated by making use of post-nonlinear independent component analysis (PNL-ICA) (Taleb and Jutten, 1999), which assumes that the observed data are component-wise invertible transformations of linear mixtures of the independence sources to be recovered.
